# Lessons learnt from 12 oral cholera vaccine campaigns in resource-poor settings

**DOI:** 10.2471/BLT.16.175166

**Published:** 2017-02-21

**Authors:** Amber Hsiao, Sachin N Desai, Vittal Mogasale, Jean-Louis Excler, Laura Digilio

**Affiliations:** a Development and Delivery Unit, International Vaccine Institute, SNU Research Park, 1 Gwanak-ro, Seoul, 08826, Republic of Korea.; b Department of Policy and Economic Research, International Vaccine Institute, Seoul, Republic of Korea.; c Department of Clinical Development and Regulatory, International Vaccine Institute, Seoul, Republic of Korea.

## Abstract

Improving water and sanitation is the preferred choice for cholera control in the long-term. Nevertheless, vaccination is an available tool that has been shown to be a cost-effective option for cholera prevention in endemic countries or during outbreaks. In 2011 the first low-cost oral cholera vaccine for international use was given prequalification by the World Health Organization (WHO). To increase and prioritize use of the vaccine, WHO created a global stockpile in 2013 from which countries may request oral cholera vaccine for reactive campaigns. WHO has issued specific guidelines for applying for the vaccine, which was previously in short supply (despite prequalification for a second oral vaccine in 2015). The addition of a third WHO-prequalified oral cholera vaccine in 2016 is expected to increase the global stockpile considerably and alleviate supply issues. However, prioritization and best use of the vaccine (e.g. how, when and where to use) will remain challenges. We describe 12 past oral cholera vaccine campaigns, conducted in settings with varying burdens of cholera. These case studies illustrate three key challenges faced in the use of the oral cholera vaccines: regulatory hurdles, cold chain logistics and vaccine coverage and uptake. To pave the way for the introduction of current and future oral cholera vaccines, we discuss operational challenges and make recommendations for future research with respect to each of these challenges.

## Résumé

Améliorer l'accès à l'eau et à l'assainissement est le meilleur moyen de lutter contre le choléra à long terme. Néanmoins, la vaccination s'avère être un outil accessible et rentable pour la prévention du choléra dans les pays où cette maladie est endémique ou pendant des épidémies. En 2011, l'Organisation mondiale de la Santé (OMS) a présélectionné le premier vaccin anticholérique oral à faible coût destiné à un usage international. Afin de favoriser et de hiérarchiser l'usage de ce vaccin, l'OMS a créé en 2013 une réserve mondiale auprès de laquelle les pays peuvent demander des vaccins anticholériques oraux et mettre en œuvre des campagnes réactives. L'OMS a publié des directives spécifiques pour demander ce vaccin, qui n'était auparavant disponible qu'en quantité limitée (malgré la présélection d'un second vaccin oral en 2015). L'ajout, en 2016, d'un troisième vaccin anticholérique oral présélectionné par l'OMS devrait permettre d'augmenter sensiblement les réserves mondiales et d'atténuer les problèmes d'approvisionnement. Il restera cependant à traiter les questions de la hiérarchisation et du meilleur usage du vaccin (par ex., comment, à quel moment et à quel endroit l’utiliser). Nous décrivons ici 12 campagnes de vaccination orale contre le choléra qui ont été menées dans des régions diversement touchées par cette maladie. Ces études de cas illustrent trois grands défis qui se posent lors de l'utilisation de vaccins anticholériques oraux: les obstacles règlementaires, la logistique de la chaîne du froid et la couverture ainsi que le taux de vaccination. Afin de préparer l'introduction de vaccins anticholériques oraux, existants et futurs, nous examinons les difficultés opérationnelles et formulons des recommandations concernant de futurs travaux de recherche sur chacune de ces difficultés.

## Resumen

La mejora del agua y el saneamiento es la opción preferida para el control del cólera a largo plazo. Sin embargo, la vacunación es una herramienta disponible que ha demostrado ser una alternativa rentable para la prevención del cólera en países endémicos o durante brotes. En 2011, la Organización Mundial de la Salud (OMS) precalificó la primera vacuna anticolérica oral de bajo coste para uso internacional. Para aumentar y priorizar el uso de la vacuna, en 2013 la OMS creó una reserva global de la cual los países podían solicitar vacunas anticoléricas orales para campañas reactivas. La OMS ha publicado directrices específicas para la aplicación de la vacuna, cuyo suministro era escaso anteriormente (a pesar de la precalificación para una segunda vacuna oral en 2015). Está previsto que el hecho de añadir una tercera vacuna anticolérica oral precalificada por la OMS en 2016 aumente las reservas globales de forma considerable y reduzca los problemas de suministro. No obstante, la priorización y el buen uso de la vacuna (por ejemplo, cómo, cuándo y dónde utilizarla) seguirán siendo asuntos importantes. Se describen 12 campañas anteriores de vacunación oral contra el cólera, realizadas en entornos con distintos niveles de cólera. Estos estudios de casos ilustran los tres problemas principales que surgen al utilizar vacunas anticoléricas orales: obstáculos reglamentarios, logística de la gestión de la cadena de frío y cobertura y aceptación de la vacuna. Para allanar el terreno en la introducción de vacunas anticoléricas orales en el presente y en el futuro, se analizan las dificultades operativas y se presentan recomendaciones para futuras investigaciones con respecto a estos problemas.

## ملخص

يُعد تحسين الماء والصرف الصحي الخيار المفضل للسيطرة على الكوليرا على المدى البعيد. وبالرغم من ذلك، تُعد خدمات التحصين أداة متاحة أثبتت جدارتها كخيار فعال من حيث التكلفة للوقاية من الكوليرا في الدول التي يستوطن بها هذا المرض أو أثناء حوادث تفشي الأمراض. تم منح أول لقاح فموي منخفض التكلفة ضد الكوليرا اعتمادًا مبدئيًا للاستخدام الدولي من قبل منظمة الصحة العالمية وذلك في عام 2011. لزيادة استخدام اللقاح ومنحه الأولوية، أنشأت منظمة الأمم المتحدة مخزونًا عالميًا في عام 2013 يمكن للدول أن تطلب منه اللقاح الفموي ضد الكوليرا من أجل حملات الاستجابة للمرض. أصدرت منظمة الصحة العالمية مبادئ توجيهية محددة لاستخدام اللقاح الذي كان به نقصًا (على الرغم من الاعتماد المبدئي لقاح فموي ثانٍ في عام 2015). ومن المتوقع أن تؤدي إضافة اللقاح الفموي الثالث – الحاصل على اعتماد مبدئي من جانب منظمة الصحة العالمية ضد الكوليرا في عام 2016 – إلى زيادة المخزون العالمي بشكل كبير وتخفيف مشاكل المخزون. ومع ذلك، فإن تحديد أولوية اللقاح والاستخدام الأمثل له ستظل تحديًا قائمًا (على سبيل المثال، كيف ومتى وأين يتم استخدامه). ونوضح 12 حملة سابقة لتطعيم اللقاح الفموي ضد الكوليرا أجريت في مواقع يختلف بها عبء الكوليرا. وتوضح دراسات الحالة هذه ثلاثة تحديات رئيسية تم مواجهتها عند استخدام اللقاح الفموي ضد الكوليرا، وهي المتمثلة في: العقبات التنظيمية، والخدمات اللوجستية للنقل في حاويات مبرّدة، وتغطية خدمات التحصين واستيعابها. ومن أجل تمهيد الطريق لتقديم اللقاحات الفموية الحالية والمستقبلية ضد الكوليرا، فقد ناقشنا التحديات العملية وقدمنا توصيات للبحوث المستقبلية فيما يتعلق بكلٍ من هذه التحديات.

## 摘要

从长远看，改善水质及卫生设施是控制霍乱的优选。 不过，疫苗接种也是一种可行的方法，并且已经证实对于疾病流行国家或疾病爆发期间霍乱的预防，是一种性价比高的选择。 2011 年首批供国际使用的低成本口服霍乱疫苗通过了世界卫生组织 (WHO) 的资格预审。 为增加并优化疫苗的使用，世界卫生组织在 2013 年创建了一个全球储存处，各国可从中申请口服霍乱疫苗以开展各项响应活动。 世界卫生组织发布了申请该疫苗的具体指南，该疫苗之前一直处于短缺状态（尽管第二种口服疫苗在 2015 年通过了资格预审） 于 2016 年推出的第三种通过世界卫生组织资格预审的口服霍乱疫苗有望大量增加全球存储并缓解供应问题。 然而，疫苗的优化及最佳使用（如，如何使用、何时使用以及在哪里使用）仍将面临挑战。 我们描述了过去在遭受不同程度霍乱影响的地区开展的 12 项口服霍乱疫苗活动。 这些案例研究表明口服霍乱疫苗的应用主要面临三大挑战： 监管障碍、冷链物流及疫苗覆盖率及接种率。 为扫清当前及未来引进口服霍乱疫苗所面临的障碍，我们讨论了实际操作方面的挑战并为有关各项挑战的未来研究提出了推荐意见。

## Резюме

Усовершенствование водоснабжения и санитарии является предпочтительной мерой для борьбы с холерой в долгосрочной перспективе. Тем не менее вакцинация является доступным инструментом, который, как было продемонстрировано, является экономически эффективным вариантом для профилактики холеры в эндемичных странах или во время вспышек этого заболевания. В 2011 году первая недорогая пероральная вакцина против холеры для международного использования получила преквалификацию Всемирной организации здравоохранения (ВОЗ). Для увеличения объемов использования, а также определения приоритетного использования вакцины в 2013 году ВОЗ создала ее глобальные запасы, из которых страны могут запросить пероральную вакцину против холеры для кампаний по противодействию этому заболеванию. ВОЗ опубликовала конкретные рекомендации по применению вакцины, которая ранее была в дефиците (несмотря на преквалификацию второй пероральной вакцины в 2015 году). Ожидается, что добавление третьей пероральной вакцины против холеры, преквалифицированной ВОЗ в 2016 году, значительно пополнит мировые запасы и облегчит проблемы поставок. Тем не менее остается сложной задачей определение приоритетного и наиболее эффективного использования вакцины (например, как, когда и где использовать). Мы описали 12 прошлых кампаний с применением пероральной противохолерной вакцины, проводимых в условиях с различной микробной нагрузкой. Эти клинические наблюдения иллюстрируют три основные проблемы, с которыми сталкиваются при использовании пероральных противохолерных вакцин: нормативные препятствия, проблемы с материально-техническим обеспечением холодовой цепи и проблемы охвата вакцинацией. Чтобы подготовить почву для внедрения текущих и будущих пероральных противохолерных вакцин, мы рассматриваем эксплуатационные проблемы и разрабатываем рекомендации для будущих исследований в отношении каждой из этих проблем.

## Introduction

The World Health Organization (WHO) estimates that there are 1.3 to 4.0 million cholera cases annually and that 21 000 to 143 000 of them result in death.^1^ Additionally, in cholera-endemic countries, 1.3 billion people are at risk of cholera.^2^ The high morbidity and consequent mortality caused by cholera is attributable to several factors, including lack of access to safe drinking water, poor sanitation and poor hygiene practices (WASH).^3^ Recent estimates suggest that cholera is endemic in 69 countries, with sub-Saharan Africa accounting for the majority of cases between 2008 and 2012 (7.0 of 11.6 million; 60%), followed by South East Asia (3.4 of 11.6 million; 29%).^2^

Improving water and sanitation is the preferred choice for cholera control in the long-term. Although progress has been made towards providing universal access to piped water and water treatment,^4^ 663 million people worldwide still do not use improved drinking water sources that can reduce the spread of contaminants such as fecal matter.^4^ Sanitation is likewise lacking for 2.4 billion people, 950 million of whom still practise open defecation.^5^

Vaccination has been shown to be a cost‒effective, more immediate option for cholera control and prevention.^6^^–^^8^ Two oral cholera vaccines have been available for years, but have not been widely used due to either cost or licensing restrictions. With the availability of lower-cost options, cholera vaccine is increasingly being considered for use in endemic countries or during outbreaks. Table 1 provides an overview of oral cholera vaccines that are currently, or soon to be, available on the market. Current vaccines are two-dose inactivated vaccines. Several live oral cholera vaccines, including a single-dose vaccine that was recently approved by the United States Food and Drug Administration,^9^ are currently under consideration for future vaccination policy. A single-dose regimen would have great potential for use in emergency or epidemic situations.

**Table 1:** Characteristics of oral cholera vaccines currently licensed or pending licensing

Vaccine	Dukoral^®^**^a^**	ORC-Vax and mORC-Vax**^b^**	Shanchol**^b^**	Euvichol^®^**^b^**	Vaxchora	Cholvax^®^**^b^**
Place of initial licensing (date)	Sweden (1991)	Viet Nam (1997, 2009)	India (2009)	Republic of Korea (2015)	United States (2016)	Bangladesh (pending)
WHO pre-qualification (date)	Yes (2001)	No	Yes (2011)	Yes (2015)	No	No
Manufacturer	Developed by SBL Vaccine (Solna, Sweden); now Valneva (Montreal, Canada)	VabioTech (Hanoi, Viet Nam)	Developed by Shantha Biotechnics (Hyderabad, India); now Sanofi Pasteur India (Mumbai, India)	Eubiologics (Seoul, Republic of Korea)	Paxvax (Redwood City, United States)	Incepta (Dhaka, Bangladesh)
Additional notes	Requires buffer for administration. Difficult to use in emergency situations. Has not been widely used apart from traveller’s market. Two-dose (≥ 6 years of age) and three-dose (2–5 years of age) inactivated vaccine	Only available for Viet Nam market. Two-dose inactivated vaccine	First low-cost oral cholera vaccine with WHO prequalification for international use. Two-dose inactivated vaccine	Two-dose inactivated vaccine	First live-attenuated oral cholera vaccine composed of *Vibrio cholerae O1 (Inaba)*. Currently indicated as a single-dose regimen for ages 18–64 years	Only available for Bangladesh market. Two-dose inactivated vaccine

WHO: World Health Organization.

^a^ Composed of killed whole cells of *Vibrio cholerae* O1 (classical and El Tor biotypes) and recombinant B-subunit of cholera toxin; requires a buffer solution for administration; recommended for people ≥ 2 years of age.

^b^ Composed of killed whole cells of *V. cholerae* O1 (classical and El Tor biotypes) and *V. cholerae* O139; no buffer for administration; recommended for people ≥ 1 year of age.

In 2011 the first low-cost oral cholera vaccine obtained prequalification by WHO for international use.^10^ Prequalification certifies the acceptability of a vaccine for purchase by the United Nations Children’s Fund (UNICEF) and other United Nations (UN) agencies; the main vaccine procurers for low-income countries.^11^ In 2013, Gavi, the Vaccine Alliance approved financing of a stockpile of an oral cholera vaccine for use in endemic and epidemic settings. Although the financing (115 million United States dollars) could support a stockpile of 20 million doses over the following 5 years, full capacity could not be achieved due to a short supply of vaccine. Thus, vaccine deployment was low, despite demand for the vaccine.^12^ To help overcome anticipated supply constraints, the International Vaccine Institute facilitated the transfer of the vaccine technology to a second manufacturer, which led to WHO prequalification of a second affordable oral cholera vaccine for global use in December 2015 (Table 1). This has already begun contributing to the global stockpile of oral cholera vaccines^12^ and is projected to increase the supply significantly in 2017.^13^ The same manufacturing technology for the vaccine was transferred to a third manufacturer, who is expected to begin production of the first-ever oral cholera vaccine registered and licensed for use in Bangladesh ‒ one of the countries most affected by cholera ‒ in the near future.^14^^,^^15^

As demonstrated by the creation of the stockpile, global interest in cholera control has increased,^16^ which should help pave the way to global use, availability and distribution of the vaccine, particularly in low-income countries through the UNICEF and Gavi procurement mechanisms. It is still not known, however, what the demand would be for oral cholera vaccines. Based on experiences from other vaccines, even with increased production capacity, adoption of new vaccines into policy takes time, and actual demand may not meet projected demand. The long-term support for oral cholera vaccines will depend on impact and cost information gathered through 2018.^17^ The Gavi board will reconvene in 2018 to reconsider its oral cholera vaccine strategy for 2018–2022,^18^ which could have an impact on the future direction of oral cholera vaccination, including its financing. Moreover, an increased supply will not alleviate vaccine delivery costs, a barrier that many countries in need of oral cholera vaccines face.^19^ Understanding financing constraints on increased use of oral cholera vaccines will be critical in the coming years, but is a complex issue that is beyond the scope of our report.

Several countries have now used oral cholera vaccine in cholera-endemic settings or hotspots (preventive); cholera outbreaks or epidemics (reactive); or humanitarian emergencies (pre-emptive).^10^ Over 4.8 million doses of vaccine have been administered in over 21 vaccination campaigns globally from 2011 to 2015, mostly supplied through the global stockpile created in 2013 (Nogareda C, WHO Global Task Force for Cholera Control working group, unpublished data, December 2015).^20^

Deploying the vaccine to the right target population, at the right time and place, often in resource-constrained settings, presents many operational challenges.^10^ In this paper, we describe the lessons learnt from 12 campaigns with complete data, conducted between 2011–2015 (Table 2). We focus on the stockpile for use in emergency settings and discuss the three key operational challenges faced with the use of oral cholera vaccines: regulatory hurdles, cold chain logistics and vaccine coverage and uptake. Aside from advising Gavi and WHO, and informing other policy-related decisions, this information may provide guidance for the introduction of oral cholera vaccines in the countries most in need.

**Table 2:** Oral cholera vaccine coverage as reported by selected campaigns, 2011–2015

Year	Site, country	Setting**^a^**	Target population, no.	Campaign coverage, no. (%)**^b,c^**		Total doses delivered, no.
				First dose	Second dose	
Feb 2011	Dhaka, Bangladesh^21^	Trial, pre-emptive	172 754	141 839 (82)	123 666 (72)	265 505
May 2011	Odisha, India^22^	Trial, pre-emptive	51 865	31 552 (61)	23 751 (46)	61 919^d^
Apr 2012	Artibonite department, Haiti^23^	Emergency, pre-emptive and reactive	50 000^e^	45 417 (91)	41 242 (82)	86 659
Apr 2012	Port-au-Prince, Haiti^24^	Emergency, pre-emptive and reactive	51 814	52 357 (101)	47 520 (92)	99 877
Jun 2012	Forecariah and Boffa districts, Guinea^25^^–^^27^	Emergency, reactive	209 000^f^	172 544 (83)^f^	143 706 (69)^f^	316 250
Dec 2012	Maban county; Jamam, Doro, Batil and Gendrassa refugee camps, South Sudan^28^	Emergency, pre-emptive	143 438	130 560 (91)	128 365 (89)	258 925
Jan 2013	Mae La refugee camp in Mae Sot, Tak province, Thailand^29^	Emergency, pre-emptive and reactive	43 485	35 399 (81)	27 658 (64)	63 057
Aug 2013	Petite Anse and Cerca Carvajal, Haiti^30^	Emergency, pre-emptive and reactive	107 906^g^	113 045 (105)	102 250 (95)	215 295
Feb 2014	Minkaman, Tomping and Juba UN mission compounds, South Sudan^31^	Emergency, pre-emptive	126 000^h^	79 850 (63)	60 421 (48)	140 271
Feb 2015	Shashemene, West Arsi zone, Ethiopia (Development and Delivery Unit, International Vaccine Institute, unpublished data, July 2015)	Trial, pre-emptive	62 161	47 137 (76)	40 707 (65)	87 844
Mar 2015	Nsanje, Malawi (Development and Delivery Unit, International Vaccine Institute, unpublished data, June 2015)	Emergency, pre-emptive	160 482	156 592 (98)	109 128 (68)	265 720
Aug 2015	Nuwakot and Dhading, Nepal (Epidemiology and Disease Control Division, Nepalese Ministry of Health and Population, unpublished data, September 2015)	Emergency, reactive	10 084^i^	10 540 (105)	10 112 (96)	20 652

UN: United Nations.

^a^ Setting refers to the situation in which the oral cholera vaccine campaign was implemented: trial = a demonstration project or trial; emergency = humanitarian situation to control cholera outbreak or anticipated outbreak; pre-emptive = pre-empting an emergency; reactive = reacting to emergency; pre-emptive and reactive indicates that the campaign was initially planned to pre-emptively vaccinate, but due to various factors became reactive once the campaign was implemented. All campaigns used Shanchol^™^ (Sanofi Pasteur India, Mumbai, India) as it was the only oral cholera vaccine available for use via the global stockpile.^17^

^b^ Percentage of target population figures may be more than 100%. In some campaigns, the initial baseline target population figures were based on either government-reported data or project team baseline census data. Therefore, the target population may be an overestimate or underestimate, especially in highly mobile populations. In some cases, individuals outside the campaign catchment area also attended vaccination sites. Percentage coverage was calculated using the target population number as denominator for both first and second doses.

^c^ All coverage is actual number of people who received one and two doses, unless otherwise indicated.

^d^ In Odisha, there were an additional 6616 doses delivered to individuals who attended vaccination sites but resided outside the catchment area. This number is included in the total doses delivered.

^e^ In Artibonite department, the initial census in the target Bocozel area had fewer than the targeted 50 000 people for vaccination; thus, the area was expanded to neighbouring Grand Saline to reach this target. Census figures were not available in the published report and thus 50 000, based on vaccine availability, was used for the coverage calculation.

^f^ A household survey of 5248 people was conducted by the implementers after the campaign to assess the number of people who received first versus second doses based on self-reporting.^26^ Reported coverage was > 90% for those who received at least one dose and 76% for those who had two doses. The authors noted that differences from the vaccination records were likely due to an overestimation of the actual population size (*n* = 209 000).

^g^ In Petite Anse and Cerca Carvajal, 200 000 doses were available. Based on the two-dose coverage rates reported by the country, Petite Anse had 80 030 (92%) coverage of its population of 86 989; Cerca Carvajal had 21 754 (104%) coverage of its population of 20 917. However, this results in over 200 000 doses. The total doses delivered here were based on a calculation using the coverage rates reported in the post-vaccination cluster survey to evaluate two-dose coverage in the two areas: 699/1118 (62.5%) in Petite Anse and 621/808 (76.8%) in Cerca Carvajal. The number reported here for total delivered doses is likely to be an underestimate, as it does not account for individuals who received only one dose.

^h^ In the South Sudan February 2014 campaign the target population was based on the doses deployed (252 000) to the area. Because this was a sudden displacement of thousands following a conflict, the exact target population was difficult to estimate. As of March 2014, there were > 168 000 internally-displaced persons living in the United Nations mission compounds. There was an estimated 84 000 living in Minkamam, Awerial county.

^i^ The second-round coverage calculation used 10 540 (number vaccinated in first round) as the denominator for calculation of coverage.

## Regulatory hurdles

A major programmatic difficulty is anticipating the capacity of countries to accelerate the introduction of the oral cholera vaccine. WHO prequalification depends on the ability of the national regulatory authority where the vaccine is manufactured to oversee the vaccine quality based on monitoring of production, quality control and good manufacturing practices. Apart from critical factors such as safety and efficacy, WHO also considers the ability of the vaccine to meet programmatic needs within a country (e.g. ease of administration).

Some countries may accept the use of a vaccine based on licensing in selected other countries; others allow the use of the vaccine under UN or UNICEF procurement processes (i.e. local licensing granted on the basis of WHO prequalification); and still others enforce the need for their own regulatory process for the licensing and use in the country (particularly for a new vaccine). Thus, WHO prequalification does not automatically guarantee the licensing of the vaccine in a country, and the regulatory landscape in this respect is not homogenous.

**Fig. 1:**
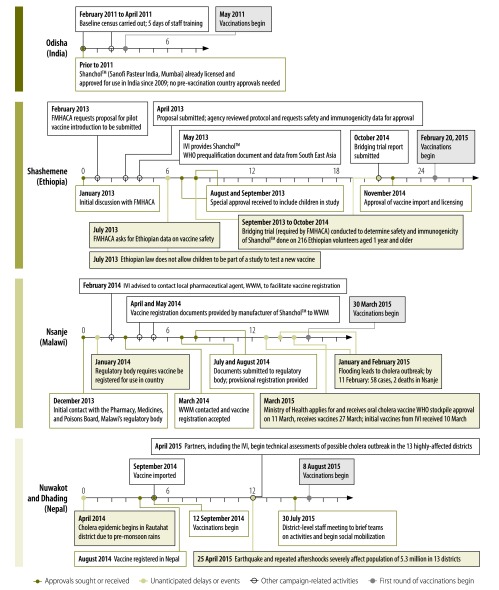
Vaccine import timelines (by month) for oral cholera vaccine campaigns conducted in India (2011), Ethiopia (2015), Malawi (2015) and Nepal (2015)

FMHACA: Food, Medicine and Health Care Administration and Control Authority; IVI: International Vaccine Institute; WHO: World Health Organization; WWM: Worldwide Pharmaceutical Malawi.

To further illustrate the circumstances faced by implementers, we describe here the regulatory process in four oral cholera vaccine campaigns where the International Vaccine Institute has been closely involved in procurement, importation and deployment of vaccine (Fig. 1). Timelines for pre-vaccination regulatory and ethical permission submissions, approvals and delays, from the initial decision to plan for mass vaccination to actual implementation, varied widely (Fig. 1).To our knowledge, other studies reviewed and included in Table 2 have not published vaccine importation and regulatory details and this is a limitation of the current literature.

Data sources: Odisha (India),^22^ Shashemene (Ethiopia; Development and Delivery Unit, International Vaccine Institute, unpublished data, July 2015), Nsanje (Malawi; Development and Delivery Unit, International Vaccine Institute, unpublished data, June 2015) and Nuwakot and Dhading (Nepal; Epidemiology and Disease Control Division, Nepalese Ministry of Health and Population, unpublished data, September 2015).

Odisha in India was the first site to use oral cholera vaccine in a mass vaccination campaign, in 2011. The vaccine had already been licensed and approved for use in India in 2009^22^ and, as such, no additional approvals or clinical data were needed.

The situation was different in Shashemene, Ethiopia, where initial discussions between the International Vaccine Institute and the Ethiopian Food, Medicine and Health Care Administration and Control Authority began in January 2013 (Development and Delivery Unit, International Vaccine Institute, unpublished data, July 2015). Protocols and available safety and immunogenicity data had to be submitted to the control authority for approval to use the vaccine, since the vaccine had been developed, licensed and WHO-prequalified in the Asian context, raising concerns about population differences. Because this was a new vaccine in Ethiopia and, by law, the country does not permit the involvement of children in biomedical research, special permission for the clinical study was required. A clinical study (bridging trial) determining the safety and immunogenicity in Ethiopian adults and children was conducted over the course of 13 months. On completion of the study and review of the data, the vaccine was approved for use in the country in November 2014, and vaccinations began roughly 3 months later, targeting individuals aged 1 year and older.

In Malawi, initial campaign planning efforts began in December 2013. In collaboration with the ministry of health and John Snow Inc., the International Vaccine Institute planned to pre-emptively vaccinate 50 000 people in Nsanje district, a particularly high-risk cholera area. Despite the vaccine already being WHO-prequalified, the regulatory authority required that it be registered in the country before use, which took approximately 6 months (Development and Delivery Unit, International Vaccine Institute, unpublished data, June 2015). Normally, full registration approval would need to be obtained from the Malawian Pharmacy, Medicines and Poisons Board, but because of the need for pre-emptive pilot vaccination before the onset of the rainy season, the board fast-tracked a provisional certificate specifically for the vaccination campaign. The provisional product registration certificate was eventually provided in August 2014 for the initial 110 000 doses planned, although lack of an adequate vaccine supply led to additional delays. On 13 January 2015, the President of Malawi declared a state of disaster in 15 districts affected by flooding in the country. The International Vaccine Institute agreed to redirect these doses to respond to the emergency, and a task force (consisting of the International Vaccine Institute, WHO, the Malawi health ministry, John Snow Inc. and Médecins Sans Frontières) was created to guide and plan the implementation of the reactive vaccination campaign. The existing vaccine import permit from the pre-emptive vaccination also facilitated the importation of additional vaccine from the WHO oral cholera vaccines stockpile in March 2015; vaccinations began on 30 March 2015. The Malawi campaign was therefore one of the quickest responses to an active cholera outbreak to date.

In Nepal, oral cholera vaccines had initially been imported into the country in 2014 for a reactive campaign that occurred 5 months after an outbreak in Rautahat district. Vaccinations for this campaign began in the same month (September), although unused doses remained in the country (Epidemiology and Disease Control Division, Nepalese Ministry of Health and Population, unpublished data, September 2015). Following the 2015 earthquake, thousands of individuals were living in camps for internally-displaced persons in Nuwakot and Dhading districts with limited access to WASH measures and medical care, and where the infrastructure was heavily damaged. The unused doses of oral cholera vaccines were successfully deployed to pre-emptively target internally-displaced persons in these two districts approximately 4 months later (in August and September 2015). Since the vaccines were already available in the country, the campaign did not face regulatory barriers to vaccine importation. All campaigns used Shanchol^™^ (Sanofi Pasteur India, Mumbai, India) as it was the only oral cholera vaccine available for use via the global stockpile.^17^

The regulatory hurdles described above demonstrate that even with a vaccine that is prequalified by WHO, i.e. meeting international standards for safety, quality and efficacy, there may be unanticipated delays to approval for use and importation. Although the WHO prequalification programme in theory reduces the need for duplicate work by a country’s national regulatory authority to import a vaccine licensed elsewhere, the reality is that some countries may require additional registration and clinical data. To address this, WHO is undertaking a major programme to strengthen national regulatory authorities by working with Member States to evaluate and improve regulatory system performance.^32^ Evidence exists that vaccine adoption by low-income countries may take as long as 20 years, and is a function of several factors: price, political will, cost‒effectiveness and feasibility within a country’s existing service delivery networks.^33^ Although international support for use of oral cholera vaccine is clearly needed, it should be emphasized to country health authorities that a multisectoral, integrated approach that is comprehensive and that also includes surveillance and diagnostics will require strong political commitment.

Even though pushing through a vaccine approval may not be the top priority of a national regulatory authority, country leaders should be made aware of the regulatory processes and typical timelines for vaccine receipt. Experience-sharing among countries may be useful in this regard, particularly to highlight possible obstacles. It is possible that countries may ease their regulatory requirements as oral cholera vaccines become used more widely and as more data on the safety and effectiveness of oral cholera vaccines in different populations are made available. However, the public health community should continue to raise awareness and identify reasons for lack of regulatory support and vaccine adoption to reduce the number of obstacles for timely introduction of oral cholera vaccines.

## Cold-chain logistics

Manufacturers recommend storing oral cholera vaccines in cold, between +2 to +8 °C, until they are administered.^34^ However, this can be close to impossible in low-resource settings, where power supplies are non-existent or unreliable, road conditions are poor and temperatures (outdoors and indoors) regularly exceed +40 °C.^22^^–^^24^^,^^28^^,^^29^ Limited capacity for storing and transporting the vaccine in continuous cold chain makes daily logistics difficult. Five of the campaigns that we reviewed (Table 2) specifically commented on cold chain difficulties in emergency settings (Research and Training Institute, John Snow Inc., unpublished data, May 2015).^22^^,^^23^^,^^25^^,^^28^

Campaigns with particularly limited ability to comply with cold chain requirements had to use what was available to maintain the integrity of the vaccine. The reactive campaign in Guinea in 2012 (Forecariah and Boffa districts) demonstrated high levels of short-term protection using a controlled temperature chain whereby vaccines were transported and delivered at ambient temperatures during the day, and unused vaccines that were returned to the cold chain at the end of the day were the first to be used the following day.^25^ In the South Sudan campaign in 2012, daily dispatches of the vaccines used cold boxes without icepacks (Jamam, Gendrassa and Batil refugee camps) or normal buckets without icepacks (Doro camp). No issues with the stability of the vaccine, as assessed with a vaccine vial monitor were reported.^28^ The outbreak response vaccination campaign in Haiti in 2012 (Artibonite department) reported that strict cold chain was challenging compared with a non-outbreak situation.^23^ However, at least two of the campaigns, in Guinea in 2012 and Malawi in 2015, documented that the vaccine quality was not affected by lack of continuous cold chain, according to the vaccine vial monitor (Development and Delivery Unit, International Vaccine Institute, unpublished data, June 2015).^26^

Growing evidence shows that oral cholera vaccines can be safely kept outside a cold chain for certain periods of time.^25^^,^^35^ A controlled temperature chain has considerable potential benefits, including cost savings and preventing vaccine damage caused by accidental freezing. More importantly, it makes it easier to vaccinate more people by allowing vaccinators to carry more vaccines at a time, thereby reducing the need to retrieve additional vaccines throughout the day. In a clinical study in Dhaka, Bangladesh, the vaccine was found to be stable at elevated temperatures (up to +42 °C) for up to 14 days, and the safety and immunogenicity in study patients were similar to those in the control group who received vaccines kept at the recommended temperature.^35^ Further studies on safety and immunogenicity in out-of-cold-chain conditions in various settings could provide more evidence for expanding the use of oral cholera vaccines. WHO has also started consultations with some regulatory agencies to clarify what type of studies are required to demonstrate when a vaccine can be safely used at ambient temperatures, prioritizing cholera and other campaign vaccines.

## Vaccine coverage and uptake

Delivering the two-dose oral cholera vaccine according to the recommended regimen presents additional challenges. Cholera vaccine campaigns must often take place concurrently with routine expanded programme on immunization (EPI) vaccinations or other public-health initiatives such as nutrition days. All the campaigns we reviewed planned their vaccination teams and site strategy (e.g. mobile, fixed, door-to-door) based on knowledge of the local context. However, vaccination administration compliance still differed widely between campaigns (Table 2).

In the Thailand campaign in 2013, vaccinations took place in the Mae La refugee camp (bordering Myanmar) where there have been recurrent cholera outbreaks since 2005. Working-age males aged 15–64 years had the lowest coverage, suggesting that future campaigns should take note of suitable times to vaccinate working-age adults. Low second-round coverage was explained by migration in and out of the camp due to seasonal work.^29^ The implementers (Thailand public health ministry and Première Urgence–Aide Médicale Internationale) also noted that high coverage in the first round may have been due to rumours in the camp that receiving the vaccination would increase the likelihood of resettlement. These circulating rumours had been dispelled by the second round.

The three campaigns in Haiti in 2012‒2013 had relatively high vaccine coverage, as compared with the other campaigns.^23^^,^^24^^,^^30^ These campaigns received support from the government as well as from local partners, such as the Group for the Study of Kaposi’s Sarcoma and Opportunistic Infections who served as the implementing partner in the Port-au-Prince campaign.^24^ The group has had a major presence in the country for more than 30 years, and their knowledge of the population and close collaborations with the Haitian Ministry of Health and Population likely contributed to its higher coverage rates in the Port-au-Prince campaign.^24^ In the campaigns in Artibonite^23^ and Petite Anse and Cerca Carvajal,^30^ strong local partners also contributed to high coverage. Similarly, in Nepal, even with vaccine delivery challenges due to natural disasters, the government improved compliance and reach by quickly galvanizing the support of female community health volunteers who play a critical role in health-care delivery (Epidemiology and Disease Control Division, Nepalese Ministry of Health and Population, unpublished data, September 2015).

Other campaigns varied in their reasons for moderate-to-low vaccine administration compliance. The South Sudan campaign in 2014 following a humanitarian crisis had low coverage compared with other campaigns in part due to overcrowding in camps. Crowding and security concerns made it difficult to administer the second dose to internally displaced persons who may have moved between camps or to other areas in the country.^31^ In the Malawi campaign in 2015, following official declaration of a disaster (Development and Delivery Unit, International Vaccine Institute, unpublished data, June 2015), the two-dose vaccination campaign was planned to reach the highest cholera risk area. However, because flooding had receded by the time of the second-round vaccination, many of the first-round vaccinated individuals had left the camp and gone back to farming activities, thus illustrating the challenges of reaching high-risk, mobile populations.

Low coverage of two vaccine doses is an issue faced by nearly every campaign that has used oral cholera vaccine. During outbreaks, particularly in the context of humanitarian emergencies where thousands of people are displaced, delivery of the second dose 14 days following the first dose is challenging. One randomized controlled trial in Kolkata, India, measured the immune response in 356 individuals who received oral cholera vaccine at the recommended 14-day dosing interval or at 28 days. There was no statistically significant difference in immune response for each *Vibrio cholerae* subtype in the two groups (*P* = 0.63–0.94),^36^ suggesting that longer dosing intervals may be just as effective. Additional studies are needed to examine whether even longer intervals are also possible. While probably not suitable for reactive campaigns, longer schedules may be particularly useful to increase flexibility of use in cholera endemic populations and internally displaced persons. However, understanding the effectiveness of longer cholera vaccination schedules will require further immunogenicity and feasibility studies.

The effectiveness of a single-dose of oral cholera vaccine is also an area for further assessment. A randomized placebo-controlled clinical study is ongoing in Bangladesh to assess the efficacy of a single dose of vaccine in 102 552 individuals compared with 102 148 who received the placebo over 6 months, 1 year and 2 years’ follow-up. The 6-month results demonstrated a vaccine efficacy of 40% against cholera overall (0.37 versus 0.62 cholera cases per 1000 persons; *P* = 0.01) and up to 63% (0.10 versus 0.26 cholera cases per 1000 persons; *P* = 0.007) against severely dehydrating cholera.^37^ Vaccine efficacy was lower (16%) in children aged 1‒4 years (among 10 211 vaccinated compared with 9765 control children). However, no published data are available on the use and effectiveness of a single-dose regimen in outbreak settings, though there may be forthcoming data from Lusaka, Zambia, where a recent outbreak occurred (April 2016) and the single-dose strategy was pursued.^38^ A modelling study of the single-dose scenario found that vaccinating more people using a single dose may avert more cases and deaths than a two-dose campaign for the same amount of vaccine deployed;^39^ observational data are still needed to further validate and support this strategy. While protection may be lower than receiving the full two-dose regimen, single-dose studies may provide evidence for use of oral cholera vaccines that reflect the realities of the targeted populations in need.

Finally, with regards to vaccine compliance, oral cholera vaccine campaigns sometimes take place while EPI vaccination programmes (e.g. oral polio vaccine and measles booster) or other community or religious activities are underway. Planners heed to consider this, to minimize interference with scheduled vaccination campaigns. For example, in India, ongoing routine public health activities caused the campaign to be cut to 3 days per round (instead of 10).^22^ Similarly in Malawi, a concurrent measles vaccination round resulted in the second round of cholera vaccination being delayed (Development and Delivery Unit, International Vaccine Institute, unpublished data, June 2015). At present, no evidence exists about an interaction between oral cholera vaccines and other orally-administered vaccines,^40^ although in South Sudan during the Minkaman campaign in 2014, the second dose of oral cholera vaccine was co-administered with the conjugate meningococcal A conjugate vaccine (further data regarding safety or immunogenicity are not available).^31^ Previous studies have demonstrated that immunogenicity is not compromised when the oral rotavirus vaccine is co-administered with the oral polio vaccine,^41^ although the two-dose oral cholera vaccine is a killed oral vaccine that is unlikely to interfere with the routine vaccinations currently administered in existing programmes.^40^ Clinical data need to be generated to address any concern of immunological interference.

## The path forward

The arrival of new oral-cholera vaccine manufacturers onto the global market will ease supply issues, but additional regulatory support and evidence on novel uses of the vaccine would aid the introduction and delivery of vaccines to countries in need. Countries’ demand for oral cholera vaccine is anticipated to increase, but it will be important to continue identifying policies that facilitate improved acceptance and distribution of the vaccines. Determining the right time and place to use vaccines will continue to be an area of focus as more evidence is collected. Overcoming cold chain and vaccine administration compliance issues will require strong local public health infrastructures and the expertise and support of public health officials. Meanwhile, innovative uses of the vaccine should be tested in the field and rigorously assessed.

Unfortunately, it is often not until an outbreak occurs that concentrated efforts are taken to quickly contain transmission. Oral cholera vaccines may provide a short- to medium-term path to a more comprehensive control package that integrates essential cornerstones of cholera prevention and control, such as the strengthening of WASH measures and effective treatment and surveillance systems. In the long-term, improving WASH will be critical to eliminating cholera, but in the meantime, additional planning needs to be made and consideration taken for using oral cholera vaccines as a complementary measure. We do not yet have a firm grasp on the most effective way to integrate the two measures, but by continually collecting and reviewing the evidence on oral cholera vaccines use, we may better understand the appropriate balance of investments into WASH, oral cholera vaccines and other interventions.
